# Descriptive epidemiology of childhood cancer in Cali: Colombia 1977-2011

**Published:** 2013-09-30

**Authors:** Luis Eduardo Bravo, Luz Stella García, Paola Collazos, Paula Aristizabal, Oscar Ramirez

**Affiliations:** 1 Registro Poblacional de Cáncer de Cali, Pathology Department Universidad del Valle. Cali, Colombia; 2 Rady Children`s Hospital San Diego, CA USA; 3 POHEMA Foundation, Centro Médico Imbanaco de Cali Colombia Cali Colombia

**Keywords:** Child, neoplams, epidemiology, incidence, leukemia, lymphoma, mortality, survival

## Abstract

**Aim:**

The objective of the present report is to describe the occurrence and survival patterns of childhood cancer over the last 20 years in Cali.

**Methods:**

Information was obtained from the Cancer Population Registry in Cali and the Municipal Department of Health . Childhood cancer international classification was used. The vital status was obtained from MDH death certificate and hospital databases. Additionally, clinical records were revised and, in some cases, telephone contact was carried out. Follow-up was done until 31/12/2011. Incident and mortality rates were estimated and adjusted for age. Life-tables were made to estimate overall survival.

**Results:**

Between the years of 1977-2011, there were 2311 cases identified in children under 15 years of age. The IR and MR for Cali were found to be 141.2 and 55.6 per million of people per year. Leukemias, lymphomas, CNS tumors and soft tissue sarcomas showed IR of 60.1, 20.5, 25.7 and 9.4, respectively. 5-years OS was 48%, and showed an improvement from 24.9%±4.3 to 51.8%±4.6, compared 1992-96 vs 2002-06 periods.

**Conclusion:**

The IR found is comparable with those described in affluent countries. Taking into account that pediatric cancer is curable for about 75-80% of the cases, it presents an enormous challenge to the Colombian health system: to improve current clinical results.

## Abstract (es)

**Resumen:**

El cáncer infantil es poco frecuente pero representa la segunda causa de muerte en >1 año de edad en Colombia. Cada año >160.000 niños son diagnosticados con cáncer en el mundo y alrededor de 90.000 mueren por esta enfermedad.

**Objetivo:**

Describir los patrones de ocurrencia y supervivencia por cáncer infantil en los últimos 40 años en Cali, Colombia.

**Métodos:**

La información fue obtenida del Registro Poblacional de Cáncer de Cali y de la Secretaría de Salud Pública Municipal de Cali (SSPM). Se utilizó la clasificación internacional de cáncer infantil versión-3. El estado vital se obtuvo de datos de mortalidad de la SSPM y de las bases de datos de los egresos hospitalarios. Adicionalmente se revisaron las historias clínicas y en algunos casos se realizó contacto telefónico. El seguimiento se hizo hasta el 31/12/2011. Se estimaron las tasas de incidencia (TI), mortalidad (TM), se ajustaron por edad y se realizó un análisis de período para la sobrevida global (SG).

**Resultados:**

Se identificaron 2311 casos en <15 años entre 1977-2011. Las TI y TM para Cali fueron de 141.2 y 55.6 por millón de personas-año. Leucemias, linfomas, tumores del SNC y sarcomas de tejidos blandos mostraron TI de 60.1, 20.5, 25.7 y 9.4 por millón de personas-año, respectivamente. La SG a 5 años fue 48%, que al discriminar por quinquenio mostró una mejoría de 24.9%±4.3 al 51.8%±4.6, al comparar los periodos 1992-96 vs 2002-2006.

**Conclusión:**

La TI encontrada es comparable con las descritas en EEUU y Europa, sin embargo, la supervivencia es menor. Dado que se ha demostrado que el cáncer infantil es curable en alrededor del 75-80% de los casos, se plantea un gran reto para el sistema de salud Colombiano, mejorar los resultados actuales.

## Introduction

Childhood cancer is an uncommon group of diseases in the population, with ASR (per million of people <15 years old per year) that fluctuate from 106 to 203^1^ ^-^ ^3^ In the American region, most cases of CC (65%) occurs in Latin America and the Caribbean, where 17.500 new cases are diagnosed each year and more than 8.000 deaths are registered because of these illnesses.^3^ ^,^ ^4^

In developed countries, CC long-term survival proportion is close to 80%.^5^ ^,^ ^6^ unfortunately, this proportion descends to 10-20% in the poorest countries of the world, in which information, opportune diagnosis and access to treatment are often difficult.^7^ The number of children with these illnesses is greater than in most affluent countries, due to the their demographic composition. The combination of these two factors makes the population impact of these illnesses relevant from the public health point of view in countries with low and medium resources.^7^ With the exception of the city of Cali, where a cancer population-based registry exists, knowledge on the risk of CC in Colombia is limited, and the population estimations of the survival of these patients is unknown. Nevertheless, national statistics from year 2008 show that CC, occupied 2nd place as the main cause of death in children and teenagers from 2 to 19 years of age.

In children, the cell origin of cancers is diverse. Compared to adults in which around 80% is epithelial, in children most of them are not^8^. Due to this a histological classification is considered more appropriate than a topographical one, which is commonly used in adults.^8^ Therefore, there was a prerequisite for the development of a classification for childhood cancer. The first one, internationally accepted, was published in 1987.^9^ At present, the one used is the ICCC in its third version, developed by the IARC.^10^ ^,^ ^11^

This classification includes 12 main tumoral groups and 47 subgroups. It is necessary to remark that this only includes tumors considered of malignant behaviour, with the exception of group III where intracranial or intra-spinal tumors of benign histology, are also included. The 12 main groups are: I leukemias, II lymphomas and reticular neoplasms, III central nervous system tumors, IV neuroblastoma and other peripheral nervous cell tumors, V retinoblastoma, VI renal tumors, VII liver tumors, VIII malignant bone tumors, IX soft tissue sarcomas, X germinal tumors, trophoblastic, and other gonadal tumors, XI other epithelial neoplasm and melanoma, and XII other specified neoplasms and malignant non-specified neoplasm.^11^

It is not possible to carry out CC community control with primary prevention activities. Neither, is there a way to make early intervention as screening strategies are not suitable, at the current time (with the exception of retinoblastoma). Therefore, the responsibility of its control falls directly upon the capacity to achieve a proper diagnostic classification, highly complex medical treatment and a strong family and social engagement.

The determination of the burden of cancer on the population can only be made possible through the availability of information about the incidence of the disease, mortality, and survival of the sick individuals. This knowledge provides a framework to contribute to the control of these illnesses. Here, we report the estimates of pediatric cancer incidence, mortality and survival in the last 40 years, in Cali.

## Materials and Methods

### Study population

Information on cancer incidence and mortality for all the tumor types in subjects <15 years of age, was obtained from the RPCC databases and the MDH,^12^ Briefly, RPCC is a cancer population-based database established in 1962 that obtains continuous information on the incidence of all cancer types of the urban population of Cali. RPCC actively collects information for the different types of cancers using as data sources both private and public health services, as well as death certificates.^12^ ^,^ ^13^

We selected as incident cases all children living in Cali from 1977 to 2011 with first malignant neoplasms diagnosed in each subject, with the exception of benign tumors of the CNS -that also were included. We used ICCC-3 classification for grouping.^11^

The RPCC complies with the international standards of good practice; it is a credited member of the World Cancer Registration Association and demonstrates exceptional quality in its information.^14^ ^,^ ^15^ RPCC shows a proportion of cancer cases with morphological verification of 94.4%, and 0.5% of cases with the death certificate as the only evidence of cancer, and a mortality incident ratio of 0.36.

### Vital status determination

We determined the vital status of children by matching: 1) the MDH mortality database (290.357 registries from 1984 to 2011), 2) with the SISBEN survey database, and 3) with the hospital/clinics records database. We included hospitals/clinics in Cali that serve as referral centers for complex pediatric pathologies such as the Hospital Universitario del Valle, the Fundación Valle del Lili, Centro Médico Imbanaco and the Rafael Uribe Uribe Clinic. We could estimate mortality rates since 1984, but for survival analyses we only had the data to include patients diagnosed since 1992. We excluded from survival analysis those cases with tumors detected during autopsy or by only means of the death certificate.

We used the ID card number as an initial step for key identification of the paired registrations. To solve the problem of cases without an ID card, we selected the following attributes for matching: first name, middle name, first surname, second surname, date of birth, direction and telephone. The process started with an exact chains comparison followed by a truncated chains comparison (three first characters), a comparison by approximation of chains and, finally, comparison by means of phonetic codes. To match the ages and dates it was necessary to take into account the absolute tolerance percentages and the daily tolerance percentages. For the matching of the information, we used a probability approximation based on the hidden Markov chain models and a deterministic approach with rules defined by RPCC based on frequency charts, dictionaries and correction lists for the names, surnames, dates, places and localities. When a couple of records where compared, the percentages of tolerance and the comparison functions provided a vector of weight for each field compared that allowed the classification of the pairs into one of three possible groups: matching, no-matching and possible matching.

We verified the reliability reviewing, manually, the medical records, the cancer morbidity surveys and the subject mortality chart records for the all the residents of Cali. By doing that verification, we could establish coincidence of each pair of cases.

We included an active follow-up using a survey of the registered cases visiting various sources of information, following RPCC standardized procedures.^12^ ^,^ ^16^ When we had no information about a case, RPCC workers helped us contacting it by means of a home visit or a phone call.

####  Outcome

The outcome variable was the death of the child. To do the analyses of survival, it was necessary to determine the time contributed by each study subject. For this estimate, we used the date of diagnosis and the date of death. If we identified a subject as alive at the end of the study period, we used that date to determine his survival time. When subject vital status at the end of the study period was unknown, we used the last contact date with vital status data. We decided to use as the date of initial observation January 1 1992, and as the completion date June 30 2012. Survival for each childhood cancer group was described in terms of 1-year, 3-years, 5-years and 10-years of observed survival. The observed survival and corresponding standard errors were computed via the life tables method in SEER*Stat.^17^ Five-year observed survival was estimated for four quinquennial periods, (i.e. 1992-1996; 1997-2001; 2002-2006, 2007-2011). The patients who were alive at the end of the study or were lost to follow-up during the study were censored.

We estimated the annual average incidence and mortality rates per million people per year. We also determined age-specific rates according to the following groups: 0-4, 5-9, and 10-14 years. For the estimation of the global rate, we did an adjustment by age taking into account the world population structure as followed by Segi. The population denominators used for calculation of the incidence rate were estimated from the Colombian national population censuses (DANE).^18^ Trends of rates were evaluated by the annual percentage change (APC), using the weighted least squeres method embedded in the US National Cancer Institute´s publicly accessible SEER*Stat software.^17^ The APC represents the average percentage of annual increase or decrease in cancer rates adjusted by age, assuming a constant rate of change for the incidence or mortality rates over the evalution period. For this, we assumed a constant rate of change for the incidence or mortality rate during the interval of time evaluated. We examined this tendency in cancer incidence using Poisson regression. This modeled the natural logarithm annual rates for age groups, as a function of the year of diagnosis. From the corresponding regression coefficient of these models, we derived the APC and its 95% confidence interval.^17^

We defined as censored observations those children who remained alive at the end of the study period or were lost during follow-up. We used the life-table method to determine survival probabilities. We estimated the standard error using the Greenwood method. For all the analyses, we considered a two tailed *P-value *<0.05 as significant. We carry-out data analyses in the statistical program Stata 10.0. 

## Results

During the period of study, 2.311 incidence cases were registered, of which 54.5% were males and 40.2% by those younger than 5-years-old. If these percentages are discriminated by five-year periods, there are no substantial changes in how much the cases occurred according to age or gender.

The method of diagnosis was morphological in 95.0% of the cases (histological 63.9%, hematology 29.3% and cytology 1.7%), and 4.5%, clinical diagnosis (including different imaging methods). For the analysis of survival, 8 (0.5%) cases were excluded for having the death certificate as the only evidence available.

The distribution of neoplasm's, according to the ICCC-3, is presented in Tables 1 and 2. Most of the cases (69.6%) concentrated on the first three main diagnosis groups: leukemias (group I 37.3%), lymphomas (group II 15.4%) and CNS tumors (group III 16.3%). It is necessary to note that Neuroblastoma represented 2.8% and Retinoblastoma 3.7%. The IR was 141.2 for all the diagnostic groups. The IR values oscillated according to periods (5-year periods) and sex from 130.1 to 185.3 in boys; and 107.9 to 157.3 in girls. The age-specific rates (ASR) per group and diagnostic subgroup, sex and five-year period are presented in Tables 1 and 2. 

**Table 1:**
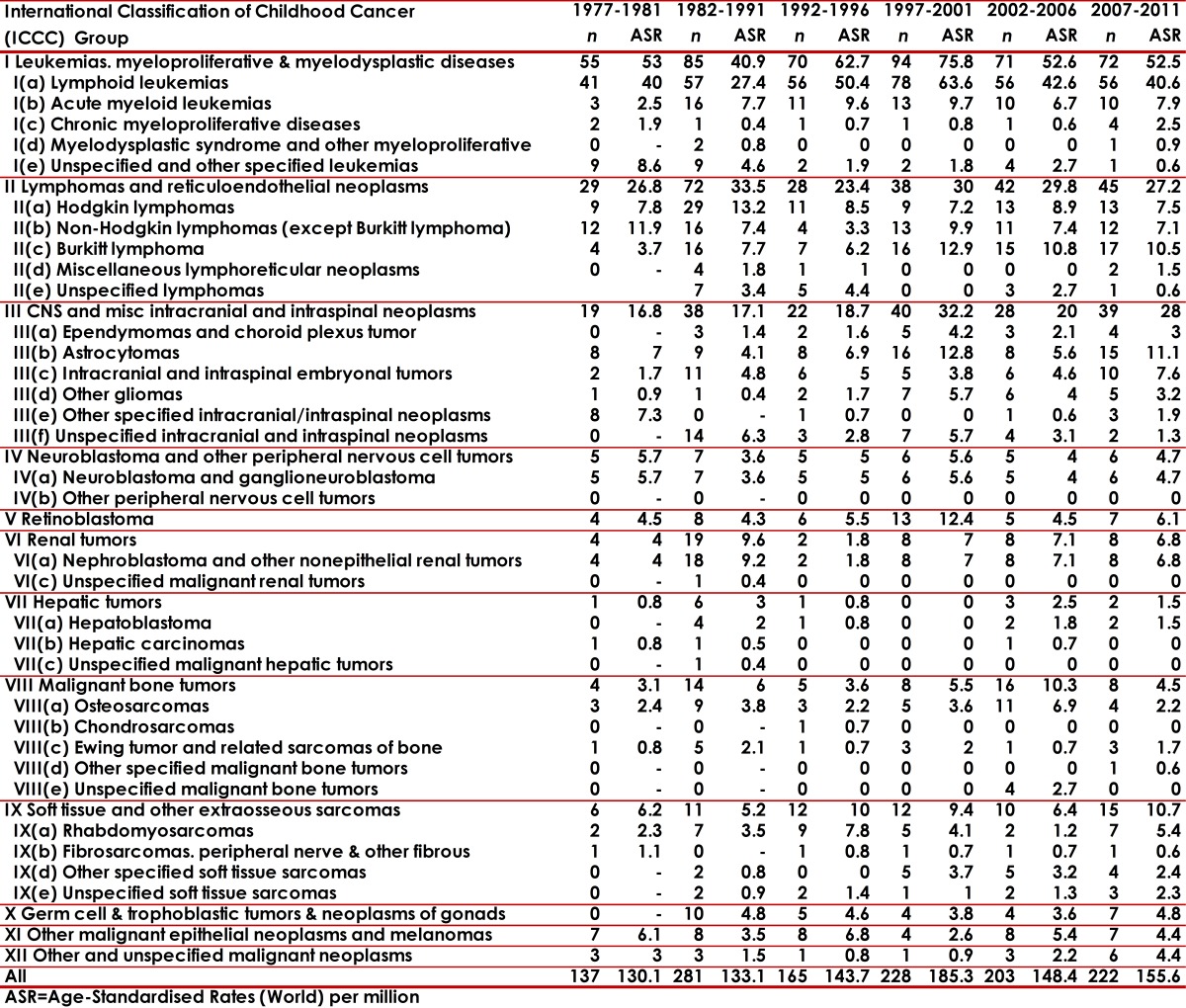
Cali, Colombia. Childhood Cancer Incidence rates per million by ICCC groups in boys from 1977 to 2011.

**Table 2:**
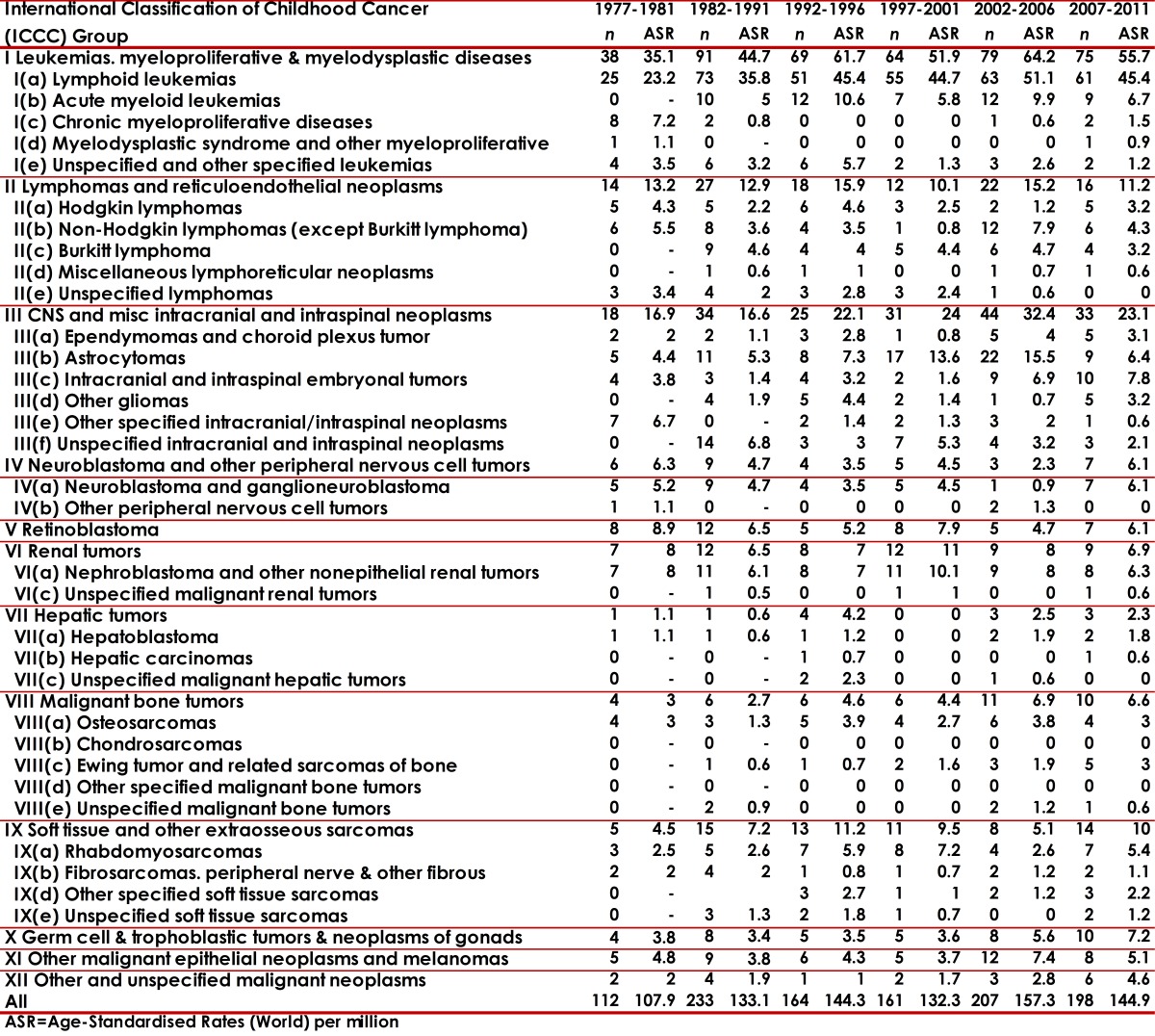
Cali, Colombia. Childhood Cancer Incidence rates per million by ICCC groups in girls from 1977 to 2011.

During the first period, the MR cause by CC for a million people per year was 66.2 and 52.9 during the years 2007-2011 (Table 3). There was a significant increase CC incidence in Cali between 1977 and 2011, with an annual percentage change (APC) of 0.9 (95% CI: 0.4, 1.5). It is remarkable to find a significant increase for leukemia with an APC of 1.0 (95% CI: 0.2, 1.8). A mortality standardized rate decrease was observed during this period in approximately 1.1 cases per year on average (Table 4). 

**Table 3:**
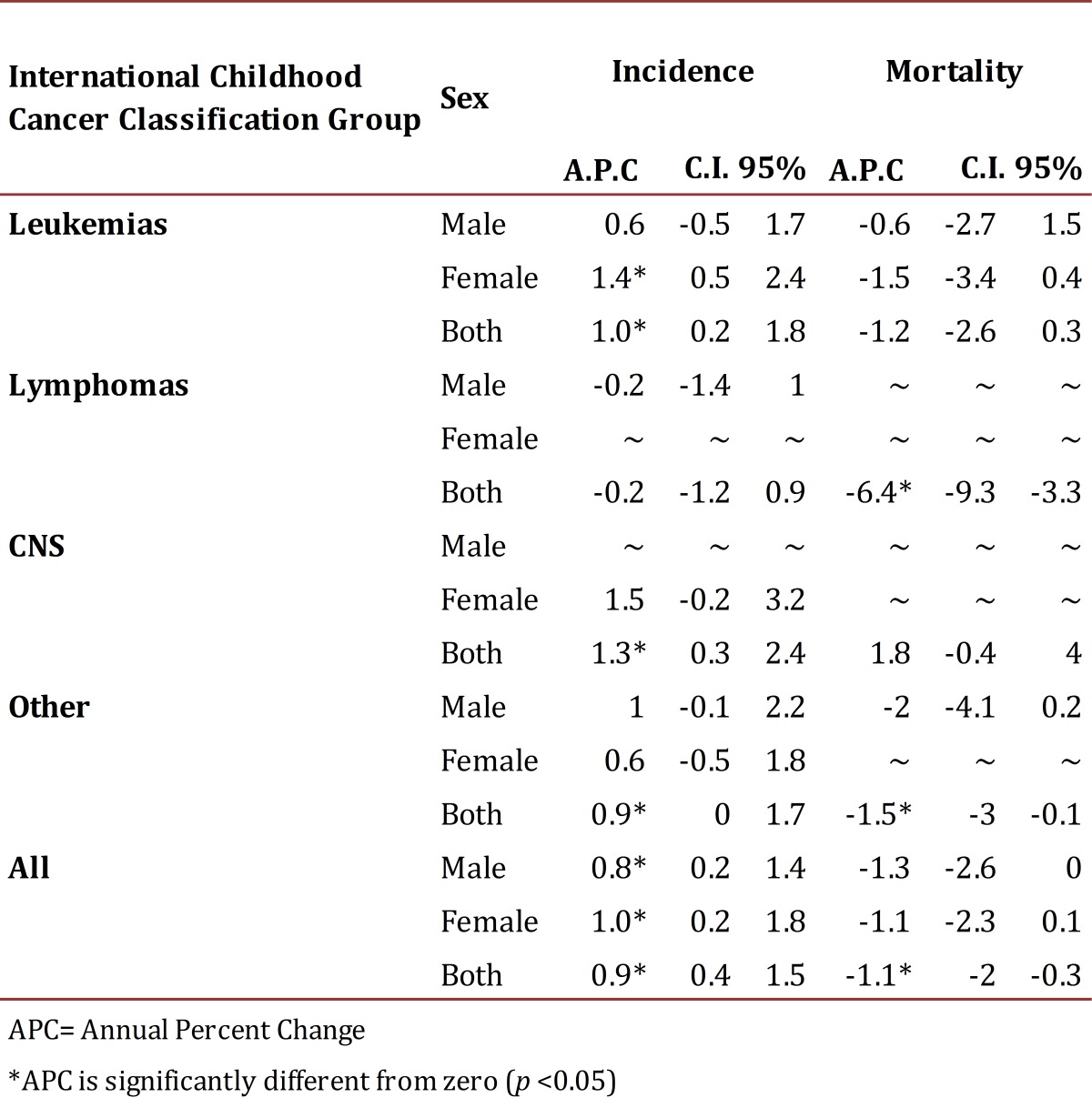
Cali, Colombia. Trends in Childhood Cancer Incidence and Mortality from 1977 to 2011

**Table 4:**
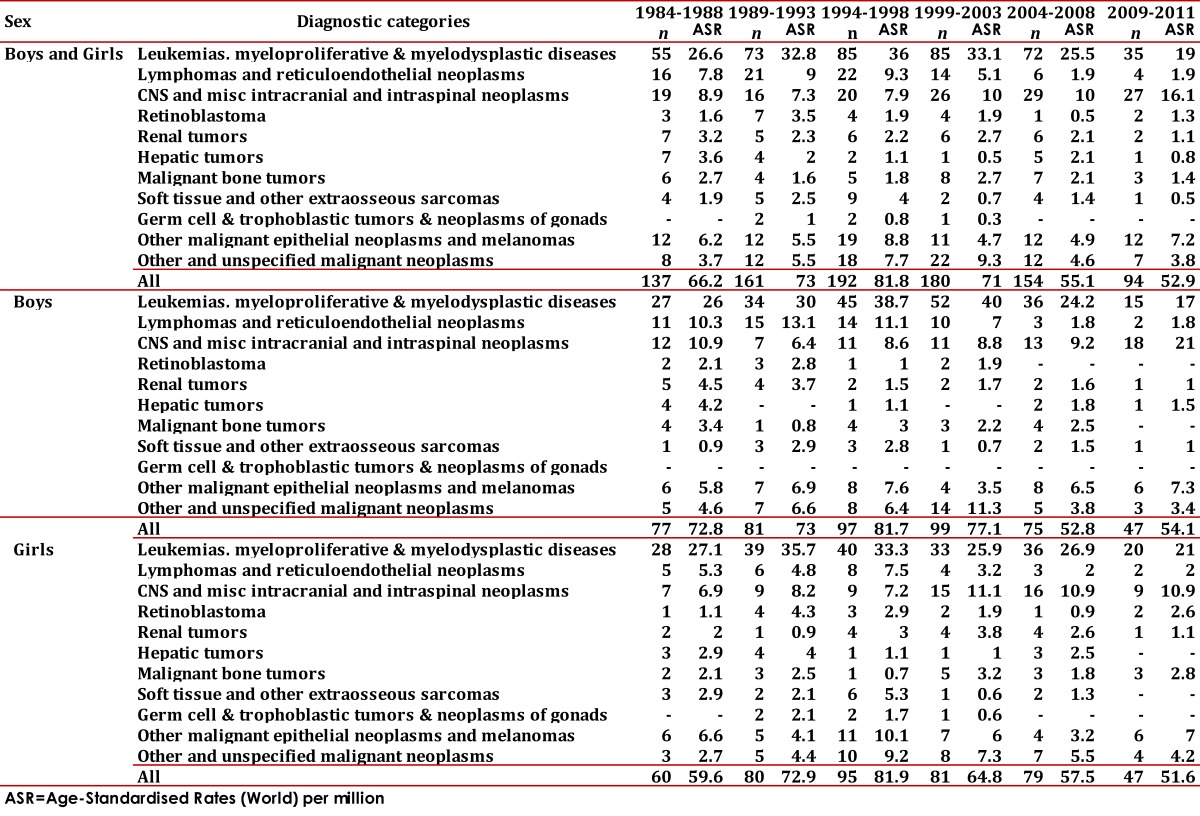
Cali, Colombia. Childhood Cancer Mortality Rates per million by diagnostic category and sex from 1977 to 2011.

The 5-year OS for the different periods was; 31.6±3.2%, 48.3±2.7%, and 54.9±2.8%. For the last five-year period, the OS was not possible to determine, but the OS at 2 and 3 years are remarkably similar to the prior five-year period (Table 5).

**Table 5:**
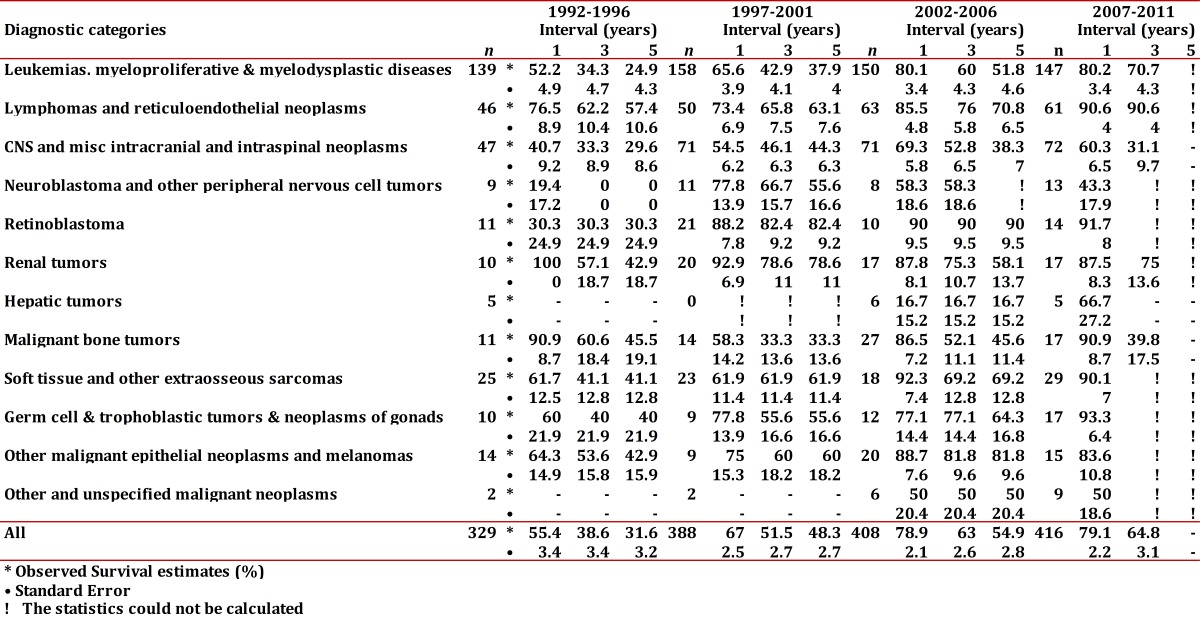
Cali, Colombia. Observed Survival estimates at 1, 3 and 5 years for Childhood Cancer trough 1992-2011 with follow-up to June, 2012.

**Figure 1:**
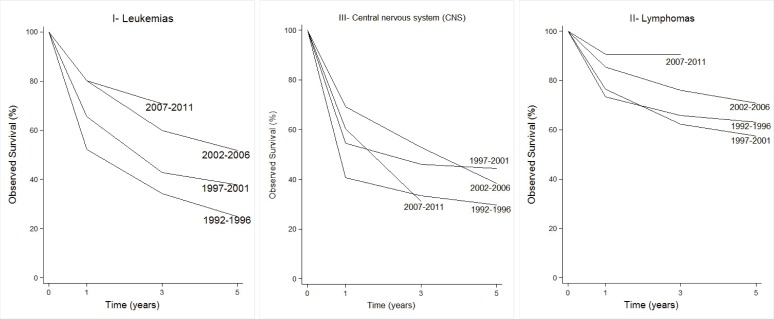
Cali, Colombia. Observed Survival estimates at 1, 3 and 5 years for Childhood Cancer trough 1992-2011 with follow-up to June, 2012

The survival for CC observed by the main diagnostic categories is shown in Figure 1. Hematological and lymphoid neoplasms (groups I and II) showed evident improvements of survival in the recent 5-year periods. In children with leukemia, the 5-year OS was 24.9% (1992-96) compared to 51.8% (2002-06). For lymphomas, the behavior was similar, and the OS rose from 57.4% to 70.8%. Although, it has not yet been possible to determine this proportion for the last 5-year period, survival at 2 and 3 years is very similar to that of the prior 5-year period. The CNS neoplasms continued showing a poor prognosis, with 5-year OS less than 50%. 

### Group I (Leukemias)

For group I the average annual IR varied from 87.9 in children <5 years old to 35.4 in the group of 10 to 14 year-olds. Among all neoplasms of this group, (ALL) was the most frequent one (82.5%). It showed a higher IR in children younger than 5 years of age for a rate of 73.5, compared to other age groups. In ALL, the group of males from 5 to 9 years old presented a greater IR than females (65.9 vs 41.0), and this is not the same for the other age groups. The ALL standardized age IR was 6.8 times greater than that of the non-acute lymphoid leukemias (AML). AML also showed a greater rate in the group under 5 years of age. Upon examining the rates of the 5-year period for this group, there is an apparent descent in the rates after the year 2002. This decreasing trend was more evident for ALL and the non-specified leukemia subgroup. The APC for this group was -1.0 (95% CI: -2.5, 0.6). The MR standardized by age for this group was of 2.5 (x 100000 /year); 2.9 for males and 2.2 for females giving a male/female ratio of 1.3. The lowest MR (2.0) was seen in under <5 years of age. The 5 and 10-year OS of children with leukemia was of 40.1±3.1% and 30.8±3.7%, respectively. Five-year OS by period (1992-1996, 1997-2001, and 2002-2006), showed the following values; 24.9%±4.3, 37.9%±4.0 and 51.8%±4.6, respectively (Table 5).

### Group II (Lymphomas)

The IR for group II was of 20.5 greater in males than in females (27.6 vs. 13.2). Among this group, Burkitt's lymphoma showed the highest IR (7.2). This lymphoma showed its highest IR in the 5 to 9 year-olds group (11.9). Most of the cases (94%) occurred in children <10 years old. In general, the rates did not show differences in the group of <5 years of age, but in the group from 5 to 9 year-olds the male/female ratio was of 2.9. Other non-Hodgkin lymphomas (excluded Burkitt's) presented an IR of 5.2, with the highest IR among children 10 to 14 year-olds (7.4). Hodgkin lymphoma showed a IR of 6.6, with an IR in the group from 10 to 14 years of 13.0. The male/female ratio for this tumor was of 2.6 and 3.7 in the group from 10 to 14 year-olds. In the last 5-year period, a decrease in the group of non-specified lymphoma was encountered. The MR for lymphomas was of 0.5 and by gender was of 0.7 for males and 0.4, for females. Lymphomas 5-year OS was 65.4±5.2% reaching a plateau about 48 months after diagnosis. Five-year OS, by period, showed the following values; 57.4%±10.6, 63.1%±7.6, and 70.8%±6.5, respectively (Table 5).

### Group III (CNS tumors)

The IR for group III was 25.7. Astrocytoma presented the highest IR in all age groups (11.2). In males, Astrocytoma IRs were greater in children <10 years of age, than in the older group. This contrasts with females who show relatively stable rates by age group. The primitive neuroectodermal tumor subgroup, in which Medulloblastoma is included, showed a relatively stable rate in all the groups of age, with an IR of 2.8. The IR was greater for males than for females (3.9 vs 2.8). The IR for non-specified intracranial/intraspinal tumor group was of 4.0. MR of this group was 7.0, for males 6.6 and females 7.3. OS was 41.9±5.0 and 33.6±6.4 for 5 and 10 years, respectively. Five year OS by period were 29.6%±8.6, 44.3%±6.3, and 38.3%±7.0, respectively (Table 5).

### Other groups

In group IV, Neuroblastoma presented an IR of 3.8, with a greater incidence (8.8) in <5 year-old group, without substantial gender difference. OS reached a plateau at 48 months in 35.4%. Retinoblastoma presented an overall IR of 5.8; it was of 18.2 in children <5 year age group, and was higher in males than in females (6.7 vs 4.8). OS of this tumor showed notable progresses when distinguished between five-year periods.

Group VI, including Wilms tumor, Clear cell sarcoma and Rhabdoid tumor presented an overall IR of 6.2. The risk for the occurrence of these tumors was higher among females than males (8.4 vs 3.9). Under 5-year age group showed the higher incidence (11.9) for this group of tumors, and MR was of 0.2. Five and 10-year OS were 69.9±9 and 61.9±10.8, respectively. Although there was an improvement in 5-year OS comparing period 1997-2001 with the previous one (78.6 vs 42.9), this trend was not maintained in the subsequent period as shown in Table 5. In group VII, Hepatoblastoma presented an IR of 0.2; all cases occurred in children less than 5 years old.

Tumors in group VIII showed an IR of 6.2, with a higher IR in the oldest group. They showed a higher IR in males compared to females (7.5 vs 4.8). Of this group, the highest IR was for Osteosarcoma (4,2). It was also found to be higher in males than in females (5.1 vs 3.1). Ewing sarcoma presented an IR of 1.4. Overall MR for this group was of 2.0, and by gender 2.1 and 1.8, for males and females. The OS of these tumors stabilized in 29.1±10.1 at 48 months from diagnosis.

Group IX comprised soft tissue sarcomas and presented an IR of 9.4, showing similar distribution by gender. Among this group, the most frequent tumor was Rhabdomyosarcoma with an IR of 5.0. This IR showed two peaks, one in children younger than 5 year old (6.3) and other among the 10 to 14 year-olds (7.0). This tumor showed a greater incidence in females than in males (6.0 vs 3.9). Five and 10-year OS for this group were 64.1±8.7 and 45.6±13.1.

Germinal tumors in group X presented an IR of 4.4 with two peaks of occurrence; one in children younger than <5 years old (6.3) and the other, in the 10 to 14 year old group (11.0), without any major difference among genders. OS steadied at 36 months of diagnosis in 47.4±13.0.

The epithelial cancers, in group XI, presented few numbers of cases with an IR of 3.8. The age group with higher IR was that of the 10 to 14 year olds. The adreno-cortical carcinoma presented an overall IR of 0.2, with 1 female case <5 years old. Thyroid carcinoma showed a global IRof 1.0 which presented, mainly, in the 10 to 14 year old group. This was more prominent in females than in males (1.2 vs 0.8). Five-year OS for this group was 61.4±12.6. The last group showed an overall IR of 1.0.

## Discussion

We estimated an overall CC IR in Cali, during the period of study, of 141.2 million children (<15 years old), ranking us in an intermediate place in comparison with other countries. In year 1998, IARC reported IR from 160.0 (Hispanics in Los Angeles) to 122,1 in England and Wales. Most recent information from Europe, according to the ACCIS system, reported an overall IR of 138.5, which varies according to the region from 131.1 in the United Kingdom to 160.1 in the northern countries (1). ^6^ ^,^ ^19^ ^-^ ^21^ The U.S. SEER reported an overall IR of 154. Brazil reported IR which oscillated between 94.7 (Salvador Bahia) and 226.2 (Goiania). This geographical variation is important and suggests the presence of environmental factors that contribute in the causality of these tumors. It is also necessary to keep in mind, in order to interpret these changes in the IRs the role that sub-registration problems and classification errors may play.

In general, CC cases in Cali occurred more often in children younger than 5 years of age and in males; this with the exception of Wilms tumor, thyroid cancer and rhabdomyosarcoma, in which the male/female ratio was of 0.46:1, 0.66:1, and 0.65:1. The predominance of the Wilms tumor and thyroid carcinoma in females is consistent with reports from other countries. Nevertheless, the female predominance of rhabdomyosarcoma does not show as much consistency and, for example, in the ACCIS and the SEER, the ratio between male/female for this tumor was of 1.3:1. ^6^ ^,^ ^19^ ^-^ ^21^

As was previously mentioned, the most frequent group was that of the leukemias, followed by CNS tumors and then lymphomas. This pattern of occurrence is similar to those reported in North America and Europe. In other South American countries, a higher frequency of lymphomas, over CNS tumors was reported, especially in Brazil. In Africa, excluding the southern part of this continent, the tumors with more IR are lymphomas, especially Burkitt's. This tumor is considered endemic of this region and is believed to be due to the coexistence of high malaria transmission and early infection by EBV. ^6^ ^,^ ^19^ ^-^ ^22^

ALL showed its greatest incidence rates in children younger than 5 year old for both genders. Nevertheless, it showed a high peak of incidence in the group of males 5 to 9 years old, compared with that of females. This is compatible with what has been described in other countries, which corresponds to the peak of incidence of common precursor B-cell cell leukemia, usually with t(12;21), occurring in males around 5 years-old. Looking at the Cali's IRs for 1977-1981 period, the IR is approximately equal for males and females (37.8 vs 37.9), suggesting that this characteristic peak of incidence of this illness did not occur before 1981. Upon taking the 5 to 10-year OS of this illness, the existence of a great gap with most developed countries is evident. Nevertheless, it is encouraging to found that there is an apparent progressive increase of 5-year OS, which is practically double between the first and third five-year period, reported in this study.

In the epidemiology Burkitt's lymphoma, attention is focused on the enormous differences that are encountered according to geographical area. In equatorial Africa, for example, Burkitt's lymphoma is considered endemic and IR of 36.1 has been reported (Uganda, Kampala 1992 to 1995), compared with those reported in U.S. Afro-descendants (SEER 1983-1992) with an IR of 0.6. The south and central American countries report IRs among 7.2 (Cuba 1986 to 1990) to 0.5 (Costa Rica 1984 to 1992). In Cali (1982-1991), an IR of 6.2 was published, and during this recent reporting period there was a rate of 7.2. This is the only lymphoma that is more frequent in younger (<5 year old) age group, occurring almost 3-times more frequently in males than in females. The other non- Hodgkin lymphomas and Hodgkin lymphoma were found to be more frequently in the older age group (10 to 14 years old) than in younger ones. Lymphoma was the tumor group that showed the best 5 to 10-year OS, with a clear increasing trend comparing each five-year period. In the last period, more than two-thirds of those patients were cured. ^6^ ^,^ ^19^ ^-^ ^21^

CNS tumor^23^ group was second in frequency and the first one in MR. Although 5 to 10-year OS for all this group was similar to that of the leukemia group, when examining 5-year OS by time periods, CNS showed no prominent increases, as were found for leukemia and lymphoma groups. In Cali, Neuroblastoma IR (3.8) is comparatively lower than in other countries and only represents 2.8% of all registered tumors children. In North America, Europe and Australia, Neuroblastoma represents between 6% and the 10% of all CCs, with an IR that varies between 7 and 10. In the Central American countries and Hispanic of Los Angeles, IR was reported to be less than 6.0. We found that the majority of cases (74%) occurred in children younger than 5 year old. Retinoblastoma, in Cali, represented 3.9% of all the cases, which is similar to reports from North America, Europe and Australia (2% to 4%). In contrast with Brazilian reports, where IR as high as 9.8 was reported in Natal city (Rio Grande do Norte state). Although the 5-year OS found for Retinoblastoma is not bad, it would be the hope to find higher OSs, given that it is a tumor susceptible for early detection and has effective therapeutic interventions. In spite of the previous mention, an important increase of the 5-year OS, from 30% to 90% was noted between the period of 1992-1996 and 2002-2006. Renal tumors represented 4.3% of the total, and in this group Wilms tumor was the most frequent. We considered that the incidence found for these tumors in Cali (6.2), is intermediate (from 6 to 9) compared to other reports. In Asia, including India and Japan, the incident of these tumors is 2 to 4. Wilms tumor long-term OS is considered as a paradigm in the treatment of CC as quickly improvements were obtained by the usage of combined surgical, chemotherapy and radiation therapies. We found 5 and 10-year OS below those reported in other countries. ^6^ ^,^ ^19^ ^-^ ^21^ ^,^ ^24^

The uncommon Hepatoblastoma is mostly an infant cancer, representing only 0.3% of all CC reported for this period in Cali. This frequency is relatively lower compared with North America and Europe where it represents about 0.8% to 1.3%. In Japan and Thailand it is higher and represents around 2.5%, been the highest IR reported in China, with an IR of 4. ^6^ ^,^ ^15^ ^-^ ^17^ Bony tumors represented 4.2% of all the registered cancers. They are tumors that usually are present in adolescence and whose frequency is rare in <5 year olds. Globally Osteosarcoma IR was 3-times greater than that of Ewing sarcoma. This relationship is similar to data reported by other cancer registries.^25^ In Asian countries, Osteosarcoma presents IR from 1 to 2, in most of the European countries and the group classified as whites in the United States the IR from 2 and 3.5, were reported. Unfortunately, the OS for this group of tumors is still bad and relatively short. We noted that this is one of the groups which did not show progress in OS, in Cali. Soft tissue sarcomas group is extensive, and highly heterogeneous, with Rhabdomyosarcoma being the most frequent one. This group represented 6.4% of all the registered tumors, in this report. The IR in different parts of the world oscillates among 2 to 5. In this group of tumors, an increase in 5-year OS was apparent, from 41.1±12.8 to 69.2±12. . ^6^ ^,^ ^15^ ^-^ ^17^ ^,^ ^26^ Germinal tumor group also includes a remarkably heterogeneous series of tumors. This group represented 3% of all the records. According to various cancer registry reports, frequency of this group oscillates between 2% and 4%. Epithelial cancer is less frequent in children than in adults, and alone they represent 2.6% of all pediatric cancer. Among this group, the unspecified carcinomas were the most frequent followed by thyroid carcinoma. We found an increase in 5-year OS of 42.9±15.9 to 81.8±9.6. ^6^ ^,^ ^19^ ^-^ ^22^

Cancer descriptive epidemiology is an indispensable source of information, which does not only offer knowledge of the causal etiologies of these illnesses, but also, contributes as an indicator of the progresses of the public health politics and the health system.
